# Supplementary material for: Nuclear Myosin 1 links genomic architecture to adipose tissue remodeling, metabolic inflammation and obesity in mice
Source: Cell Death Dis. 2026 Feb 26;17(1):270. doi: 10.1038/s41419-026-08525-3 (PMC13004839; doi:10.1038/s41419-026-08525-3)
Supplement: Supplementary file 1 — Supplementary material [file 41419_2026_8525_MOESM1_ESM.docx]

**Supplementary material**

**Supplementary Figure 1. NM1 regulates adipogenic transcriptional networks and metabolic programming independent of food intake.** (A) Observed versus predicted gene expression values for all genes included in the transcription factor activity (TFA) model. The red dotted line indicates the ideal fit (R² = 0.63; Pearson r = 0.80), demonstrating the predictive strength of the inferred network. (B) Transcription factor–target gene regulatory network inferred by integrating single-cell RNA-seq and bulk RNA-seq data using the Inferelator framework. Red and blue edges indicate predicted positive and negative regulatory interactions, respectively. Green nodes represent target genes; red nodes represent transcription factors. (C) Daily food consumption measured over 8 days in individually housed WT (green) and NM1 KO (red) female mice (n = 3 per group). No significant differences in food intake were observed between groups across the measurement period. (D) Body weight measurements of the same mice taken daily over 9 days. (E–I) Heatmaps showing gene expression patterns from RNA-seq of eWAT across WT and NM1 KO mice, grouped by functional pathway. Panel (E) shows genes involved in positive regulation of fat cell differentiation; (F) negative regulation of fat cell differentiation; (G) insulin signaling; (H) insulin secretion; and (I) endocrine resistance. The color scale represents Z-score values, with green indicating higher and red indicating lower relative expression.

**Supplementary Figure 2. Validation of adipocyte differentiation by FABP4 and PPARγ expression and Oil Red O staining.**
(A) Western blot analysis of *FABP4* and (B) *PPARγ* (protein levels in WT, HET, and NM1 KO MSC derived adipocytes at day 0 (pre-differentiation) and day 20 (post-differentiation). β-Actin was used as a loading control. Molecular weights are indicated on the right. (C) Representative images of WT and KO cells stained with Oil Red O at day 0 (pre-differentiation) and day 20 (post-differentiation) showing accumulation of neutral lipid droplets in mature adipocytes. Scale bar = 100 µm.

**Supplementary Figure 3. Western blot analysis of adipogenic markers in WT, HET, and KO MSC-derived adipocytes.** (A) Representative Western blot showing FABP4 protein levels in differentiated MSCs from WT, HET, and NM1 KO mice. β-Actin was used as a loading control. A non-specific band was observed at 30 kDa.

(B) Representative Western blot showing PPARγ protein levels in the same samples. β-Actin served as a loading control.
