# Supplementary figures and images for: Nuclear Myosin 1 links genomic architecture to adipose tissue remodeling, metabolic inflammation and obesity in mice
Source: Cell Death Dis. 2026 Feb 26;17(1):270. doi: 10.1038/s41419-026-08525-3 (PMC13004839; doi:10.1038/s41419-026-08525-3)

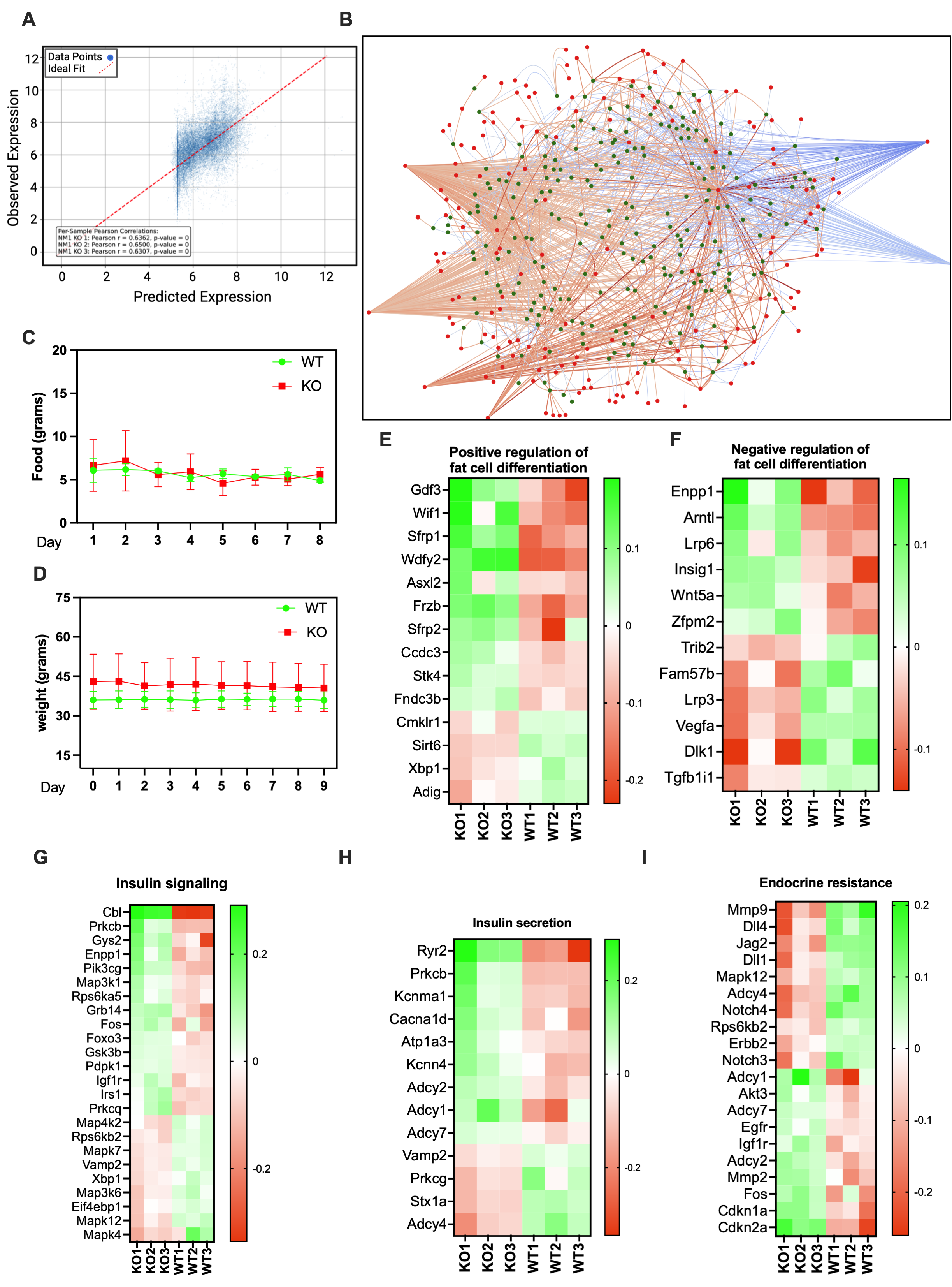

Supplement: Supplementary file 2 — Supplemental figure 1 [file 41419_2026_8525_MOESM2_ESM.tif]

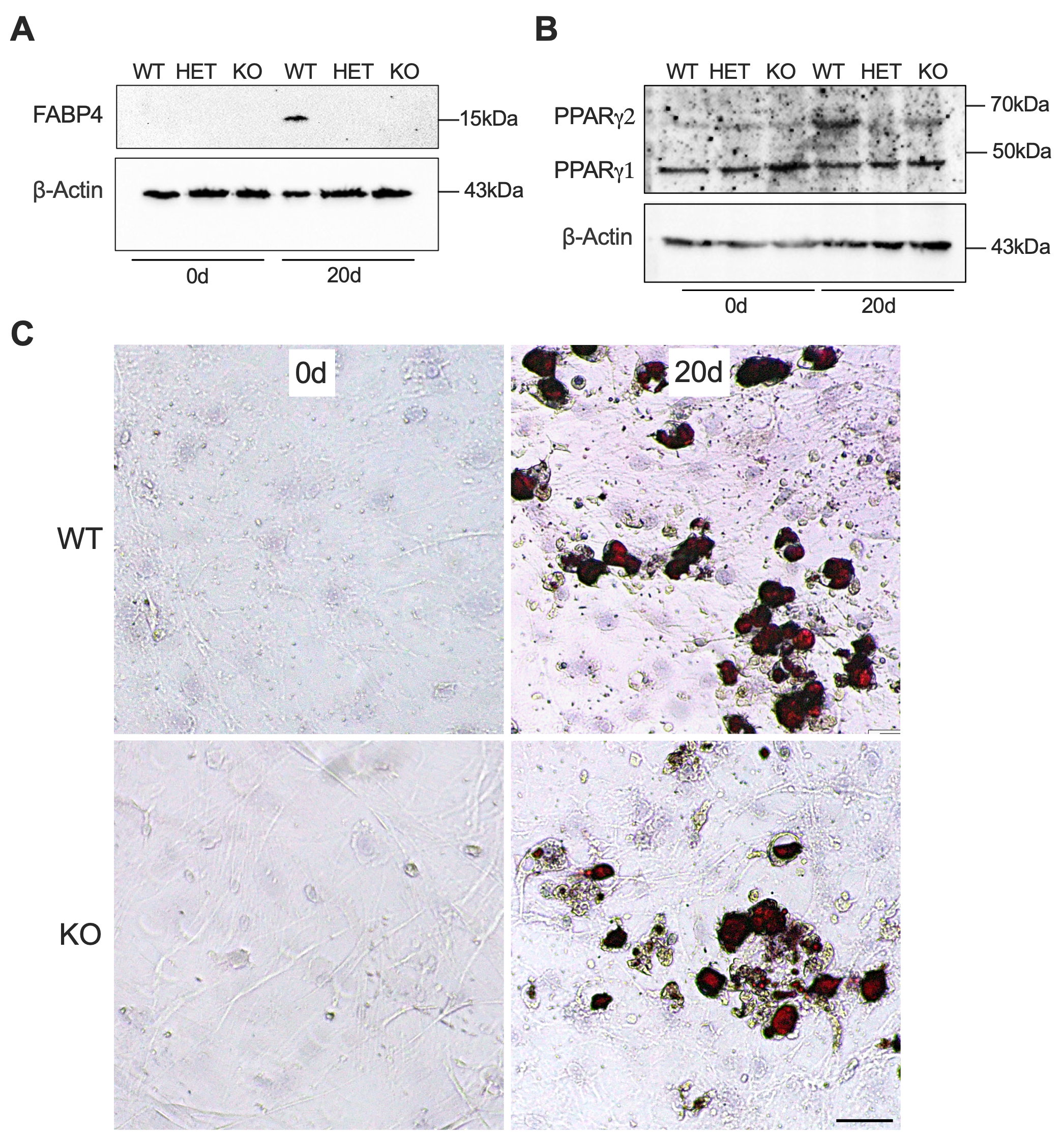

Supplement: Supplementary file 3 — Supplemental figure 2 [file 41419_2026_8525_MOESM3_ESM.tif]

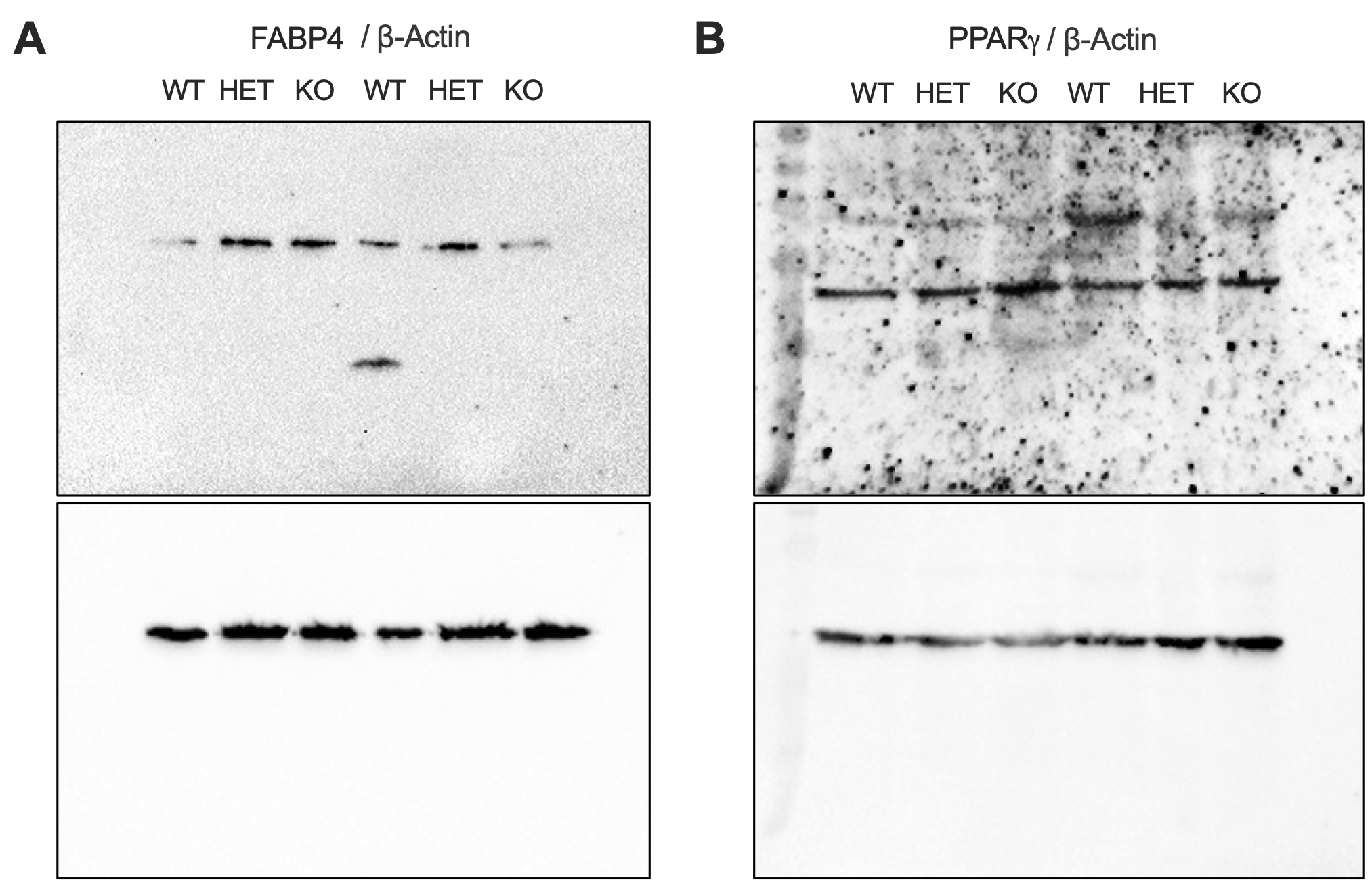

Supplement: Supplementary file 4 — Supplemental figure 3 [file 41419_2026_8525_MOESM4_ESM.tif]
